# Advancements in Smart Wearable Mobility Aids for Visual Impairments: A Bibliometric Narrative Review

**DOI:** 10.3390/s24247986

**Published:** 2024-12-14

**Authors:** Xiaochen Zhang, Xiaoyu Huang, Yiran Ding, Liumei Long, Wujing Li, Xing Xu

**Affiliations:** Department of Industrial Design, Guangdong University of Technology, Guangzhou 510006, China; xzhang@gdut.edu.cn (X.Z.); 2112317043@mail2.gdut.edu.cn (X.H.); 2112317010@mail2.gdut.edu.cn (Y.D.); 2112317082@mail2.gdut.edu.cn (L.L.); 3119008036@mail2.gdut.edu.cn (W.L.)

**Keywords:** visual impairment, wearable assistive devices, sensory substitution technology, narrative review

## Abstract

Research into new solutions for wearable assistive devices for the visually impaired is an important area of assistive technology (AT). This plays a crucial role in improving the functionality and independence of the visually impaired, helping them to participate fully in their daily lives and in various community activities. This study presents a bibliometric analysis of the literature published over the last decade on wearable assistive devices for the visually impaired, retrieved from the Web of Science Core Collection (WoSCC) using CiteSpace, to provide an overview of the current state of research, trends, and hotspots in the field. The narrative focuses on prominent innovations in recent years related to wearable assistive devices for the visually impaired based on sensory substitution technology, describing the latest achievements in haptic and auditory feedback devices, the application of smart materials, and the growing concern about the conflicting interests of individuals and societal needs. It also summarises the current opportunities and challenges facing the field and discusses the following insights and trends: (1) optimization of the transmission of haptic and auditory information while multitasking; (2) advance research on smart materials and foster cross-disciplinary collaboration among experts; and (3) balance the interests of individuals and society. Given the two essential directions, the low-cost, stand-alone pursuit of efficiency and the high-cost pursuit of high-quality services that are closely integrated with accessible infrastructure, the latest advances will gradually allow more freedom for ambient assisted living by using robotics and automated machines, while using sensor and human–machine interaction as bridges to promote the synchronization of machine intelligence and human cognition.

## 1. Introduction

Assistive technology (AT) is a broad field of study that enables people with visual impairments to overcome various physical, social, infrastructural, and accessibility barriers [1]. A central area in the field is the development of assistive technology for people who are blind or partially sighted (BLV). According to the World Health Organization, it is estimated that blindness affects approximately 39 million people; additionally, 246 million people have low vision [2]. Visual impairment prevents affected individuals from visually accessing a wide range of information about their surroundings, affecting their ability to participate in a range of social and community activities [3], resulting in barriers including reduced independence and safety, as well as education, learning, and employment difficulties. A range of different types of assistive devices for the visually impaired have been developed to overcome these barriers, including electronic travel aids (ETAs), electronic orientation aids (EOAs), hybrid assistive devices (HADs), and ADL devices. The development of smaller and increasingly powerful computing hardware permits the design of portable and wearable assistive technologies [4]. Currently, wearable assistive devices for the visually impaired are becoming an increasingly popular direction. Compared with other handheld or portable assistive devices, wearable devices have the advantage of integrating more invisibly into the lives of the visually impaired. Wearable assistive devices enable visually impaired people to free their hands and support the simultaneous use of other assistive devices (e.g., canes and guide dogs). The advent of innovative electronic hardware, materials, algorithms, and artificial intelligence technology has enabled the development of sophisticated wearable assistive devices for the visually impaired. These devices integrate a multitude of advanced functions while maintaining a lightweight, low-power, and highly efficient design, offering users optimal comfort and performance. When wearable assistive devices are developed as part of the personal attire of visually impaired people, they take on fashionable and aesthetic attributes. This allows assistive devices to serve visually impaired people more imperceptibly, thereby fulfilling their desire to integrate into the population. This is of great value in promoting social inclusion and social equity.

This study employed bibliometric techniques to examine the evolution, trends, and focal points of wearable assistive technology for visually impaired individuals over the past decade. On the basis of the study of hotspots over the past few years, we found a gradual shift in research focus from fundamental technological development to more integrated optimization and the exploration of user needs. The most recent research hotspots include multimodal perception and integration technologies, intelligent navigation systems based on deep learning, and interaction design, with the objective of enhancing the user experience. Furthermore, emerging research areas have attracted attention, including the application of interdisciplinary innovative materials, improvements in social acceptance, and addressing aesthetic and emotional needs that extend beyond functionality. However, existing reviews predominantly focus on technical classifications, operating principles, and implementation pathways, with relatively limited discussion on interdisciplinary technological innovation and sociocultural aspects.

This study addresses the following three key areas of research: (1) latest design results in haptic and auditory feedback; (2) application of smart materials in this direction; and (3) conflicting interests of individuals and society regarding the wearing of assistive devices for the visually impaired in public places. It also explores the opportunities and challenges associated with these three directions and makes suggestions for the design of wearable assistive devices based on sensory substitution technology. The design recommendations are provided as a reference for related researchers.

## 2. Materials and Methods

All data from the literature used in this study were obtained from the Web of Science (WoS) core database, and the data were retrieved using an advanced search in WoS. The final search formula was as follows: TS = ((wearable* OR “portable device” OR “portable technology” OR “body-mounted device” OR “body-worn device”) AND (“blind people” OR “blind person” OR “visually impaired” OR “visually challenge” OR “visual impair” OR “visual challenge” OR “visual impairment” OR “visual impairments” OR “vision impaired” OR blind OR blindness). The search was conducted on 18 July 2024, and the search parameters were narrowed to articles published between 2014 and 2024 and written in the English language. The following sources were excluded from the search: review articles, conference abstracts, editorial material, and news items. The remaining articles were filtered, resulting in a total of 1057 articles. Because the basic keywords for wearable assistive devices for the visually impaired always appear in the author keywords and abstracts, searching by title (TI) alone would have missed a large number of articles. Therefore the bibliometric search was performed on the subject (TS) rather than on the title (TI). Subsequently, the literature data were imported into EndNote v2.0 for de-duplication, and articles not related to the study topic were excluded by manually reading the titles, keywords, and abstracts; if there was any uncertainty in the relevance, the full text was searched for exclusion, resulting in a total of 533 articles related to the study topic.

This study presents a narrative review based on bibliometrics. The initial stage of the study employed CiteSpace, a Java-based system with broad accessibility, to detect and visualize emerging trends and transient patterns in the scientific literature [5]. This study employed bibliometric methods to examine the literature on wearable assistive devices for the visually impaired. These methods include an analysis of author cooperation, literature co-citation, keyword co-occurrence, and keyword clustering. This analysis provides a clear understanding of author cooperation, the most influential authors, research hotspots, and the evolution of research frontiers in this field. The second half of the article, however, initiates the narrative by focusing on one of the more innovative research hotspots, i.e., wearable assistive devices for the visually impaired based on sensory substitution technology.

## 3. Results

### 3.1. Overview of the Results of the Bibliometric Analysis

The following parameter settings were applied in the CiteSpace analysis: the time span was from January 2014 to June 2024; the time slice length was one; the subject term sources were fully selected by default; the thresholds were maintained as system defaults; and the paths were simplified using the keyword path method.

#### 3.1.1. Analysis of Annual Publications

**Figure 1:**
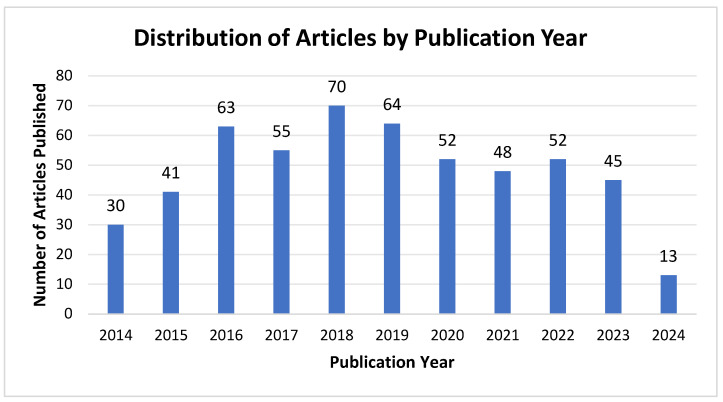
Annual publications on wearable assistive devices for the visually impaired in WoS from 2014 to 2024.

The research can be divided into two distinct phases, as shown in Figure 1. The first phase, spanning 2014 to 2018, is characterized by a gradual growth trend, reaching a peak of 70 articles in 2018. During this period, wearable assistive robotics was still in its nascent stages, with the advent of pioneering solutions, such as intelligent navigation systems and obstacle detection systems, contributing to the observed growth. The second phase is from 2018 to 2023. Since 2018, the number of publications has exhibited a downward trend, with studies still being published but at an overall decreasing rate. This decline may be attributed to the saturation of research in the field or a shift in focus towards other areas. Around 2018, research on basic technologies began to stabilise and the focus of related technologies shifted to the application and optimization phase. Early results entered the commercialization phase, research hotspots shifted to new directions, and saturation of research in traditional fields may have led to a decline in the number of publications after 2018.

#### 3.1.2. Most Influential Journals: A Co-Citation Analysis

**Figure 2:**
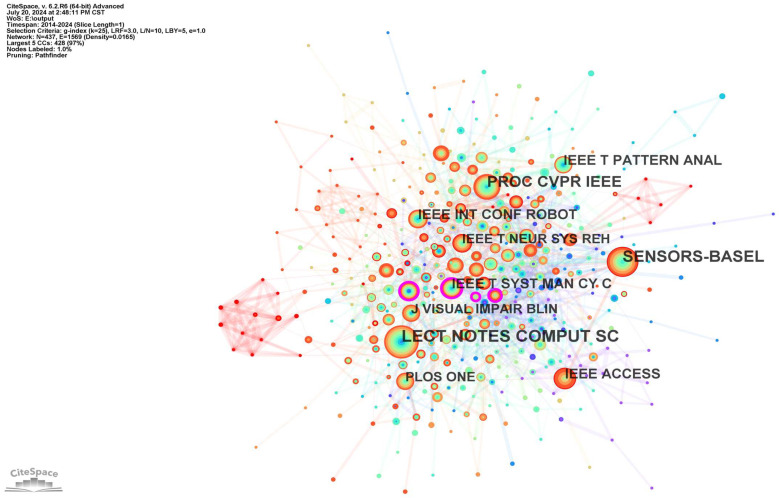
Co-citation journal map of the studied publications from 2014 to 2024.

A co-citation journal map is a valuable tool for researchers, enabling them to assess the impact and status of different journals. As shown in Table 1, in terms of the number of publications, the academic journals *Lecture Notes in Computer Science* (205) and *Sensors* (168) were the most prolific. On the other hand, *IEEE Transactions on Systems, Man, and Cybernetics: Systems* (0.11); *IEEE Transactions on Biomedical Engineering* (0.11); *Disability and Rehabilitation: Assistive Technology* (0.11); and *Proceedings of the 29th Annual CHI Conference on Human Factors in Computing Systems* (0.11) represent academic journals with a greater degree of centrality. As illustrated in Figure 2, these journals encompass a plethora of scientific disciplines, including computer science, engineering and technology, and neurology.

#### 3.1.3. Analysis of the Author Collaboration Network

It should be noted that an author’s affiliation, “Association for Computing Machinery”, was incorrectly identified as author information by CiteSpace. Consequently, the “Association for Computing Machinery” node was removed to ensure the accuracy of the author collaboration network analysis graph.

**Figure 3:**
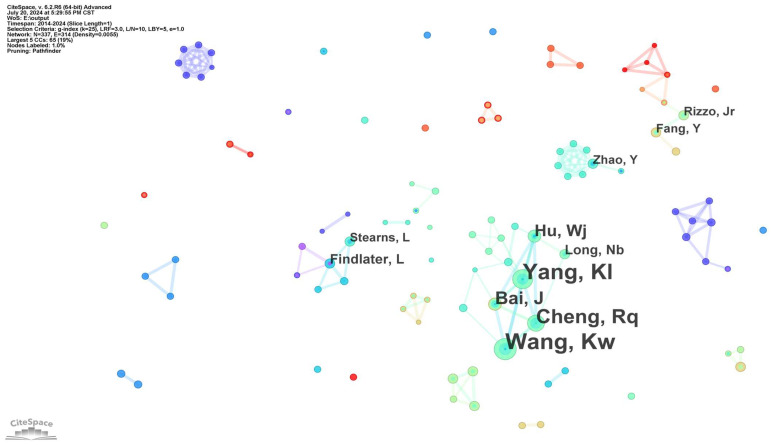
Co-authorship map of studied publications from 2014 to 2024.

Table 2 presents a list of the top 10 authors in terms of publications. Figure 3 illustrates that the field has developed a significant collective of authors centred on Wang, Kaiwei, and Leah Findlater, surrounded by numerous smaller collaborative teams with limited inter-team connections. At the same time, there are also a number of cases in which there are only two nodes with a single connectivity or an isolated node. In accordance with Price’s law, if we assume that the number of papers produced by the most productive authors in a specific field is Nmax, then M can be expressed as 0.749(Nmax)1/2. In this field, authors who have published more than M articles are defined as core authors in this study. It can be observed that Nmax is equal to 23, with an M value of approximately 3.35, rounded upward. This indicates that those who have published more than four articles are considered core authors. The statistical analysis reveals that there are 15 core authors in the sampled literature, with a total of 114 articles, representing approximately 21.39% of the total number of papers. In accordance with Price’s law, the number of papers published by these core authors is less than half of the total number of research works in this field. Therefore, it can be concluded that the core author group in this research field has not yet formed.

#### 3.1.4. Analysis of Co-Citation Reference Network

**Figure 4:**
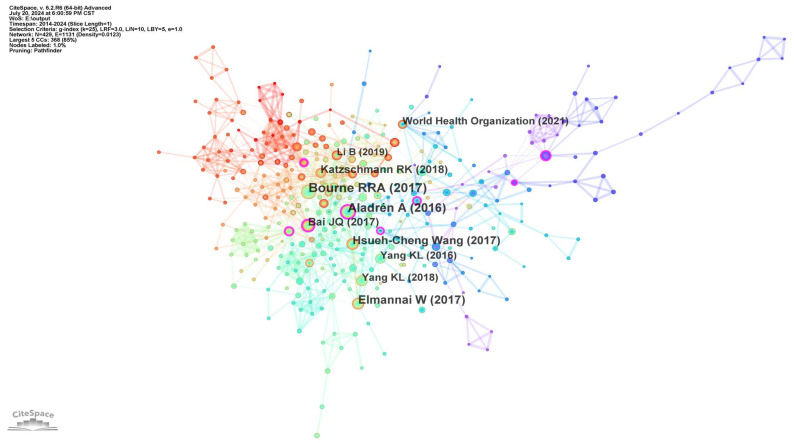
Reference co-citation map of the studied publications from 2014 to 2024.

In a co-citation reference network, consistently highly cited literature is considered as the classic literature within a field, which may reflect the developmental lineage and research foundations of the field. Table 3 lists information on the top 10 studies in terms of co-citation frequency. As shown in Figure 4, among the top three authors, Bourne focused on global estimates, trends, and projections of global blindness and visual impairment [6]. Aladren proposed a new NAVI system based on visual and range information, a system that assists or guides people with impaired vision by means of sound commands [7]. Elmannai conducted a comparative survey of wearable and portable assistive devices for the visually impaired, identifying the most important devices and highlighting their improvements, advantages, disadvantages, and level of accuracy [8].

#### 3.1.5. Keyword Co-Occurrence and Clustering

**Figure 5:**
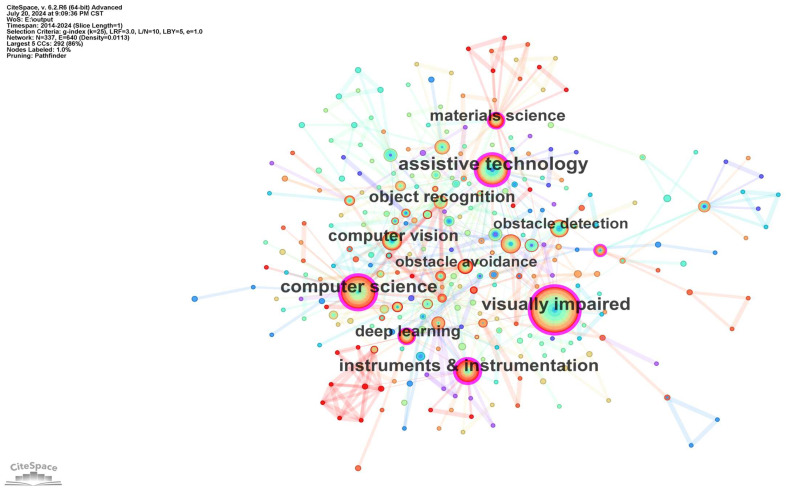
Keyword co-occurrence map of the studied publications from 2014 to 2024.

Keywords reflect the interrelationships among various topics in the literature and are a summary of the central themes of an article. Keyword analysis facilitates the study of hotspots in a field. Figure 5 illustrates a keyword co-occurrence map. Keywords with high centrality indicate significant research developments and key turning points. Table 4 and Table 5 show the top 10 keywords in terms of frequency and centrality, respectively. As evidenced in Table 4 and Table 5, a high frequency of keywords does not necessarily correspond to a high centrality. For example, the node “wearable device” has a frequency of 28, which ranks it in 6th position, yet its centrality is 0.06, which ranks it in 17th place. Consequently, it is necessary to consider both the frequency and centrality of keywords to accurately assess the research hotspots. As a synthesis of Figure 5, Table 4 and Table 5 show that, in addition to the keywords “visually impaired” and “assistive technology”, the research hotspots in this field over the last decade were “computer science”, “instruments and instrumentation”, “computer vision”, “obstacle detection”, “deep learning”, “object recognition”, “materials science”, “ultrasonic sensor”, and “wearable system”.

**Figure 6:**
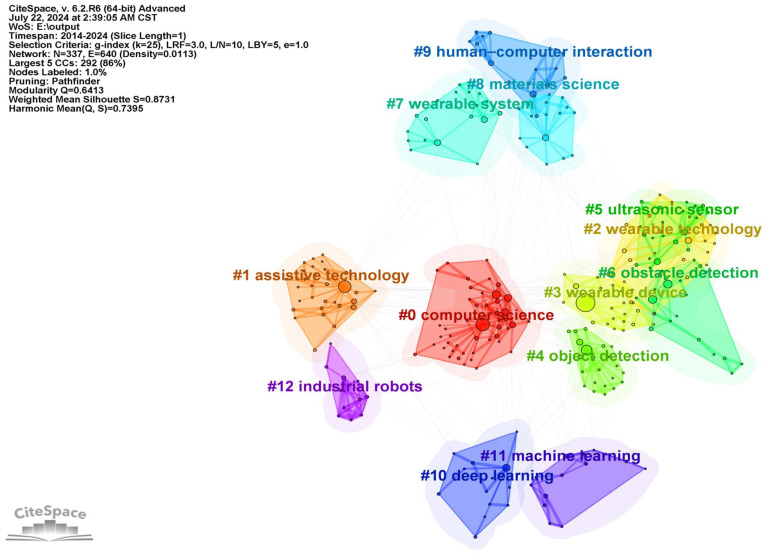
Keyword clustering map for the studied publications from 2014 to 2024.

As shown in Figure 6, the Q-value of the clustering map of the keywords is 0.6413, which is greater than 0.3, indicating that the clustering is good, and the S value is 0.8731, which is greater than 0.5, indicating that the clustering confidence test results are good. The mapping produced 16 clustering labels, as shown in Table 6. The clusters are numbered #0–#15 and, the smaller the cluster number, the more keywords the cluster contains. The numbers of keywords in clusters #13–#15 are less than 10; the clustering was ineffective, so these four clusters are excluded from discussion. Table 6 provides details of the thirteen valid clusters #0–#12. Most of the connectors in the plot are contained within the clusters, but several cross-spacing class connectors are also present. Among them, the six clusters #0, #2, #3, #6, #4, and #10 have more connecting lines across distance classes, indicating a high degree of co-citation among these research directions, which are more similar in terms of the subject matter of their research. A synthesis of Figure 6 and Table 6 demonstrates that #0 represents the core, indicating that computer science, including fields such as algorithms, data processing, and system development, is the fundamental field of research for this device. Cluster #1 focuses on the development and application of core technologies for the device, including sensory substitution technology and assistive functions. Cluster #2 explores the design and user experience of the device. Cluster #8 focuses on innovations in smart materials and the research of new sensor materials. Finally, cluster #6 focuses on the application of perception technology in real-world scenarios.

**Figure 7:**
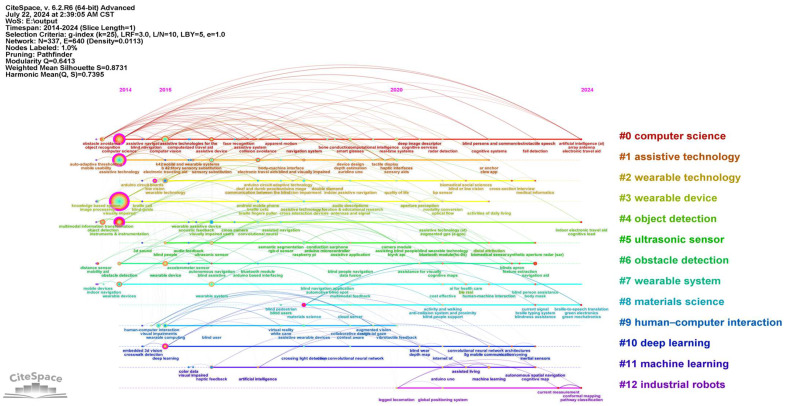
Timeline map of the studied publications from 2014 to 2024.

As shown in Figure 7, a timeline map is generated based on the keyword clustering map. As we can see from Figure 7, more hot keywords appear around 2014. This period marked the initial stage of research, with a primary focus on fundamental technology research and development, including visual perception technology [9] and obstacle detection algorithms [10]. From 2015 to 2020, there was a proliferation of both hot keywords and connections, accompanied by a rapid expansion in the technological innovation and application scenarios. The fields involved have expanded to include computer vision [11], augmented reality [12], deep learning [13], multi-sensor fusion [14], etc., with greater emphasis on technology integration and user experience optimization [15]. This proliferation can be attributed to the accelerated advancement of nascent technologies, such as the Internet of Things and deep learning, coupled with an escalating demand in the social market for assistive technologies for the visually impaired and the incremental adoption of products like smart watches and health monitoring devices. After 2020, research topics tend to mature and converge, with a decrease in the density of hot keywords and connections, but there is still some exploratory research (such as smart materials and social assistive technologies) gradually emerging. The clusters from 2014 to 2020 focused mainly on the research and development of hardware and basic technologies, while the clusters in the last five years have paid more attention to how to combine technologies such as multisensory feedback [16], artificial intelligence [17], and new materials [18] to improve device performance and user experience.

### 3.2. Wearable Assistive Devices for the Visually Impaired Based on Sensory Substitution Technology

Sensory substitution [19], which refers to the transformation of an impaired sensory form into another form perceivable by the user, has been a hotspot in research within the field of wearable visual impairment aids over the last decade. Visually impaired people suffer from visual deficits that prevent them from obtaining sufficient visual information from their surroundings to support them in performing various tasks in their daily lives. Sensory substitution technology has been widely used in wearable assistive devices for the visually impaired, i.e., replacing vision with another form of user-perceivable stimuli (e.g., auditory or tactile stimuli) to provide various forms of feedback from the surrounding environment. There are numerous studies on sensory substitution technologies, making it difficult to comprehensively discuss them all, so this study selected one of the more innovative directions as an introduction.

#### 3.2.1. Haptic Feedback Devices

Auditory and haptic systems are leading directions in sensory substitution technology, and both show promise as practical sensory substitution interfaces. Tactile–vision sensory substitution studies have been carried out by numerous research groups for over a century [20]. Haptic feedback devices use the human skin as a receptor to transmit information through various sensors, electronic devices, and mechanical elements, providing tactile information that can be felt through the skin, such as pressure, vibration, and stretching, which are transmitted to the brain through the afferent nerves [21] to help visually impaired people understand and respond to their environment. The skin is the largest organ of the body [22]; thus, there are numerous possibilities for the placement of haptic feedback devices on the body. One such possibility is a position that creates the least obstruction to other important sensory functions while potentially avoiding sensory overload by interfering with other communication mediums (e.g., hearing). Haptic feedback devices can convey simple information, such as path direction, spatial orientation, and obstacle size. Over time, they may evolve to describe more complex information [23], such as three-dimensional space [24], text, diagrams, and emotions. This enables individuals who are visually impaired to perform more complex tasks and fulfil their needs in a more comprehensive manner.

**Figure 8:**
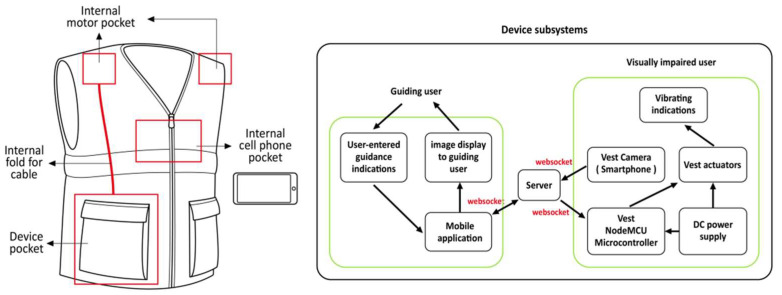
Haptic interface for remote guidance of visually impaired people [25].

**Figure 9:**
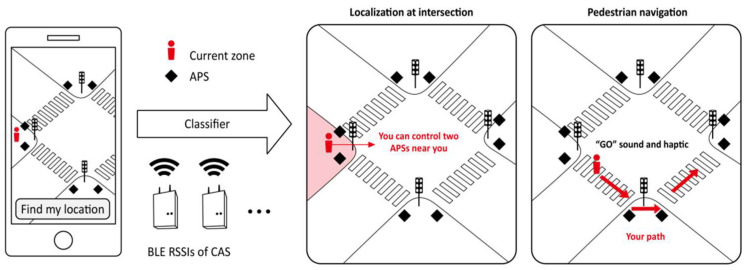
Outdoor positioning of the BLE RSSI on accessible pedestrian signals at intersections for individuals who are visually impaired [31].

Vibrotactile feedback: Vibrotactile feedback has become a widely used system because of its advantages of fast response, easy signal perception, and the miniature, inexpensive nature of the device. Figueroa-Hernandez et al. presented a classic example using a smartphone camera and the transmission of haptic instructions via a wearable vibrating device to assist individuals who are visually impaired in navigating unfamiliar environments [25]. The device has the advantages of a low-profile appearance and low cost, which can serve as foundations for further expansion. As shown in Figure 8, the device was integrated into a fabric vest. Via the smartphone’s camera and the miniature NODE MCU’s signal generator sending information to a cloud server, the remote instructor was able to obtain a real-time image of the environment of a person who is visually impaired and instruct their next move through an application developed in React Native, providing feedback in the form of vibrations. In [26], a depth camera was integrated into a hand-worn wearable device to guide individuals who are visually impaired in grasping a target object in a desktop environment by delivering orientation information for the target to the user via a vibration array feedback. Skulimowski et al. designed a wearable system consisting of a processing unit attached to the user’s chest and a haptic belt placed on the user’s abdomen [27]. The 20 vibrating actuators on the haptic band indicate the shape and distance of an obstacle through the arrangement and intensity of their vibrations. Sultania with et al. focused on the difficulties in learning STEM (diagrams, signals, graphs, etc.) for the visually impaired and an economically viable, wearable haptic Braille device was designed [28]. The device is capable of changing haptics in real time to map contextual information from a blackboard to students who are visually impaired, allowing them to overcome challenges related to perceiving spatially located alphanumeric characters, which is difficult to achieve with conventional devices. Musical haptic wearables (mhws) [29] support the potential of individuals who are blind or low vision (BLV) to learn music, and MHWs with vibrotactile alerts and vibrational changes were found to be suitable for assisting in reading music and supporting technical instruction and practice. In addition to the standalone type of wearable assistive devices for the visually impaired mentioned above, there is also a focus on the deep coupling of devices with an accessibility infrastructure. Existing solutions are mainly based on large-scale infrastructure [30], such as prefabricated routes, the placement of tags (VLC, RFID, CNF, etc.), the setup of WiFi access points, and dedicated sensors. Figure 9 shows a typical case based on accessibility infrastructure. Kiyoung et al. improved APS (accessible pedestrian signaling) using an outdoor RSSI Bluetooth to locate pedestrians [31]. Eight APS devices with Bluetooth modules were placed at road crossings, where users who were visually impaired could use their smartphones to identify their current location and plan the route and time at which to safely cross the zebra crossing, with the device providing feedback via haptics and voice.

**Figure 10:**
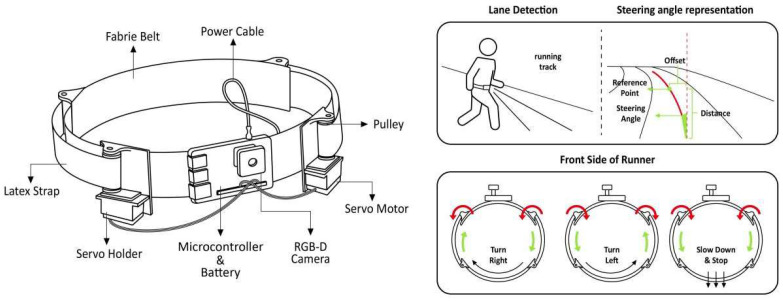
Wearable haptic guidance system based on lumbar skin stretching [32].

**Figure 11:**
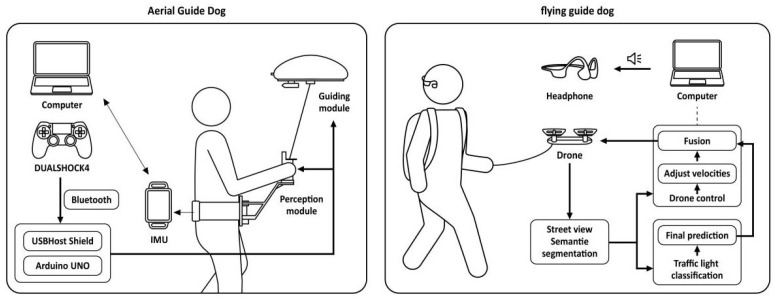
Aerial Guide Dog [33] and flying guide dog [34].

Electromechanical haptic feedback: A device transmits skin sensations, such as pressure and stretching, through various mechanical and electronic components in several parts of the body. In [32], a typical example of a wearable electromechanical haptic feedback device was presented to help individuals who are visually impaired navigate independently on a running track by providing the sensation of skin stretching. As shown in Figure 10, the system used an RGB-D camera and a microcontroller to detect the runway and calculate the steering angle for navigation, using servos to control the rotation of two latex straps that provide the haptic feedback around the waist to guide the user. Skin stretching provides a more intuitive and continuous indication of the steering angle than traditional vibration and acoustic feedback, guiding the user to more perform the appropriate maneuver more naturally without the need for excessive training. Another illustrative example of innate human innate proprioception to reduce potential cognitive load is the Aerial Guide Dog, a helium balloon aerostat drone designed for indoor navigation [33], as proposed by Zhang et al. As illustrated on the left side of Figure 11, the perception module, which is held in the user’s hand, provides a real-time traction sensation, as well as directional guidance for navigation. On the right side of Figure 11, Haobin Tan et al. focus on the assistive functions and present a prototype of a “flying guide dog” that is capable of street view semantic segmentation, recognizing traffic lights, and automatically adjusting its movement [34]. In [35], a wearable device based on light haptic cues was presented, consisting of a three-node pneumatically controlled wristband, a servo-controlled forearm attachment mechanism, and a control armband. Friendly, nonintrusive, and gentle pressure and drag sensations are generated on the user’s wrist and forearm to convey direction and distance cues. Chase et al. developed a system providing audio and haptic guidance for a user via skin stretch feedback, enabling them to explore haptic graphics on a touchscreen [36]. The system is low cost and allows for editing of the haptic guidance patterns and cues, which can be experienced remotely or reviewed independently by the user. Gandhi et al. explored the possibility of using the tongue as a practical human–machine interface, designing the TVSS as a nonsurgical, non-invasive visual prosthetic system [37].

Vibrotactile feedback has been extended from simple navigation tasks to complex tasks, such as learning music. As the demands and complexity of information transfer increase, the limitations of the inadequate information capacity of single vibrotactile feedback [38] become apparent. Achieving good spatial and semantic perceptions of a user’s environment require extensive training [39], for which the feedback must be easy to learn. Current research shows that multiple haptic actuators or the integration of other sensory feedback are often necessary to enhance the effectiveness and legibility of complex information transfer. The main challenges in this research include the limited sensitivity of the skin to tactile stimuli and the requirement for a minimum distance between actuators [40]. Further research is needed to determine optimal positions for haptic actuators and appropriate feedback types to improve information recognizability. Multimodal feedback has emerged as a potential solution to these challenges. Research combining VR and AR [41] also suggests that integrating vibrotactile feedback with other sensory modalities could significantly enhance user experience in future wearable devices.

Electromechanical haptic feedback devices provide more refined haptic feedback through the use of mechanical traction and motor drives. These devices can provide directional guidance through proprioception and reduce the cognitive load on the user [42]. However, these devices generally suffer from a number of technical bottlenecks, including loud noise [43], heat, high power consumption, bulky size [44] and discomfort [45] when worn. In particular, high power consumption and wear and tear can affect the lifetime and comfort of the device when worn for long periods. Although smart materials (mentioned in Section 3.2.3) have the potential to improve device comfort and reduce weight and power consumption, how to achieve efficient power transmission and feedback [46] remains an unresolved problem. Haptic interfaces remain on the threshold of more comprehensive development [47]. In the future, the further development of smart materials, flexible electronics and multimodal technology is expected to solve these problems and provide visually impaired people with a more efficient, comfortable and varied tactile feedback experience.

#### 3.2.2. Auditory Feedback Devices

The use of auditory feedback to provide spatial information for the visually impaired takes less training time than the use of haptic feedback and is more advantageous in terms of size and cost. Auditory feedback from wearable assistive devices for the visually impaired explores various techniques and methods to improve navigation and interaction with the environment. One approach is to use computer vision and artificial intelligence to provide real-time auditory feedback, integrating image captioning [48], face recognition [49], and depth estimation to provide visually impaired people with a comprehensive understanding of their environment. The visual alternative system proposed in [50] used auditory guidance to convert visual data into speech, conveying precise path information and obstacle cues to the visually impaired, in order to enhance the safety and situational awareness of such persons travelling independently. To enable people who are visually impaired to interact confidently with people with whom they are familiar, Sabarika et al. proposed a more integrated system that recognizes familiarity and sends personalized voice messages, while also recording conversations with strangers and providing remote monitoring and timely assistance services [51]. This enables people who are visually impaired to interact confidently with acquaintances and empowers them to make informed decisions regarding strangers. Jayakumar gave greater consideration to the social interactions of people who are visually impaired and proposed a novel speech-assisted facial emotion recognition system to enhance their understanding of others’ emotions in social interactions, as well as to help people who are blind navigate social and emotional environments more effectively [52].

**Figure 12:**
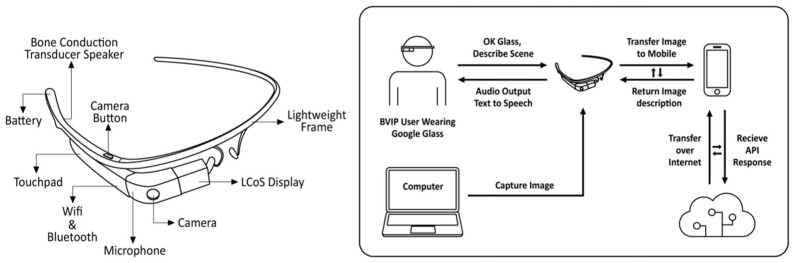
Google-Glass-based real-time scene analysis for people who are visually impaired [54].

Another approach focuses on spatial audio in virtual reality, e.g., [53], assessing the effectiveness of spatial audio and speech cues in enhancing object perception and navigation. Finally, in terms of applications, intelligent assistance systems use handy wearable devices with cameras to convert images into auditory feedback, meeting the needs of user-friendly and cognitive efficiency design. Google Glass has become an increasingly popular research topic in the field of wearable assistive devices for the visually impaired because of its attractive design, ease of wear, powerful hardware features, and ability to implement a variety of applications. In [54], a typical use case was presented, as shown in Figure 12, in which the system uses a camera embedded in smart glasses to capture images of the surrounding environment, analyzing them using a custom vision application programming Interface (Vision API) for Microsoft Azure Cognitive Services. Users can hear speech converted from the Vision API output through the smart glasses’ speakers to obtain more detailed information about a scene. The development of AI technology has further broadened the prospects for smart glasses in this area, making it possible to provide reliable and powerful real-time solutions through voice feedback. Gupta et al. proposed a smart glasses design that uses voice assistance via built-in voice assistants and sensors, which allows users to easily access internet content through AI voice broadcasting of the search results, expanding the possibilities for people who are visually impaired to handle complex tasks [55].

The main challenges in auditory feedback research are environmental adaptability, cognitive load, and user acceptance [56]. External noise in the travel environment can affect auditory feedback, and techniques such as volume amplification or adaptive equalization are required to ensure the audibility of commands [57]. The effectiveness of auditory feedback is often compromised by high cognitive demands and the need for intuitive processing. Ref. [56] points out that the high cost of auditory feedback devices and the high cognitive load caused by the difficulty of using the system can lead to reduced user acceptance. The dynamic outdoor environment requires devices that can reliably detect and communicate information about various obstacles and environmental changes, while auditory feedback may obscure important environmental sounds, leading to safety issues with navigation. Ref. [58] proposes to solve this problem through a multimodal communication framework, using other feedback to compensate for the lack of auditory feedback.

#### 3.2.3. Application of Smart Materials

As electromechanical haptic feedback devices provide natural and complex haptic feedback, they usually require many mechatronic components, leading to challenges such as large size, heavy weight, high energy consumption, mechanical susceptibility to wear and tear, and uncomfortable and difficult to wear devices. Researchers foresee low-cost, lightweight, compact, energy-efficient, and highly mobile devices capable of delivering diverse haptic feedback modalities in the near future [59]. In recent years, the application of smart materials to wearable assistive devices for the visually impaired has become a popular trend.

**Figure 13:**
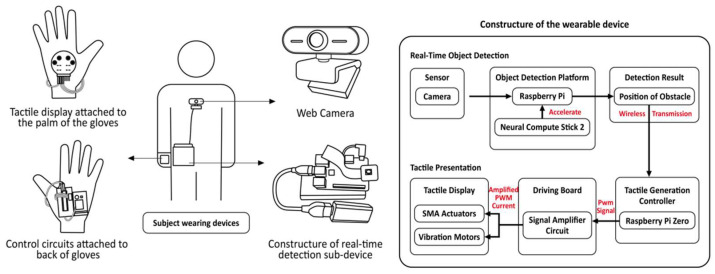
A wearable assistive device for pedestrians who are blind using real-time object detection and tactile presentation [63].

Shape memory alloys have become a relatively mature smart material in the field of wearable assistive devices for the visually impaired due to their high controllability, reliability and wide application in miniaturized actuators [60]. They can provide precise shape recovery or force feedback through temperature changes and are often used for dynamic haptic feedback and device fit adjustment [61,62] to improve user experience and device comfort. In [63], a classic case with a haptic display combining a shape memory alloy (SMA) actuator and a vibration motor is proposed, which is practical and inexpensive. As shown in Figure 13, the wearable device consists mainly of the following two sub-devices: real-time object detection and haptic information presentation. Environmental information is acquired using the camera, and afterward the compressed YOLOV3 model, deployed on a Raspberry Pi 3B+ and accelerated by NCS2, detects pedestrians in front of it in real time, and the detection results are transmitted wirelessly to the haptic presentation device based on socket communication. Although the vibration frequency of the vibration motor cannot be changed, the SMA actuator can generate vibrations with different frequencies and intensities, which can be perceived as different tactile sensations by the user. Therefore, the haptic display interface that combines the SMA actuator and the vibration motor is able to present different tactile sensations to convey obstacle information to the user. Ghodrat et al. noted the potential of SMA-based VIP haptic feedback, prototyping and evaluating the forms and parts of the body on which the haptic feedback would more likely be perceived by the user [59]. They demonstrated that a spring-form SMA wire in the form of a free-moving effector design on the skin was the most feasible and that two different types of motion, squeezing and sliding on the arm, could be effectively perceived. A wearable system is proposed in [64] that recognizes obstacles ahead of it and measures the distance in real time, suggesting safe movements via the vibration patterns of haptic gloves with SMA actuators woven into them.

Functional materials with actuation capabilities enable mechanical movement or deformation in response to external stimuli [65], providing power and motion functionality to wearable devices. In addition to relatively mature materials, such as shape memory alloys, electroactive polymers (EAPs), which undergo large deformations under an electric field [66], are being used to create lightweight and flexible haptic feedback devices to assist the visually impaired with navigation. Liquid crystal elastomers, known for their sensitivity to light or temperature stimuli, have been used in dynamic display devices to provide multimodal feedback that effectively aids Braille recognition and path guidance [67]. Sensing functional materials form the core of sensing and information acquisition in wearable devices, with flexible pressure sensors and electronic skin technologies developing rapidly in this field [68]. Liu et al. used a strategy combining flexible sensing with a memristor-based artificial neural network to design a wearable and low-cost Braille recognition system [69]. They fabricated a flexible polydimethylsiloxane (PDMS)-based pressure sensor to construct an electronic skin (E-skin) for Braille recognition. Similarly, smart fabrics based on MXene-coated yarns [70] demonstrated excellent pressure responsiveness, enabling independent sensing and high-resolution signal acquisition for tactile Braille recognition, providing innovative solutions for text learning and communication among visually impaired individuals. In the field of signal transmission and processing, materials that combine conductivity and flexibility are a major focus of research. Fully textile-based electrostatic sensors [71], integrating conductive fibers with flexible materials [72], have enabled an efficient Braille-to-speech conversion system. This system can recognize Braille in real time and vocalize it, significantly improving the efficiency of information transfer. With the growing emphasis on environmental protection, the application of eco-friendly smart materials in assistive devices for the visually impaired is gaining attention. Arbaud et al. developed the first eco-friendly wearable vibrotactile device [73], significantly reducing the proportion of non-degradable plastics in the device through the extensive use of bio-based conductive inks and biodegradable composite materials. This sustainable material innovation holds potential for large-scale production and deployment while minimizing ecological impact.

#### 3.2.4. Conflicting Interests of the Individual and Society

Social acceptance is a key factor in the widespread adoption of wearable visual impairment aids. For wearable devices to fulfil their potential and for users to adopt a particular wearable device, people must first deem the device acceptable for themselves and others to wear [74].

For individuals, acceptance of a device is influenced by a combination of factors, such as functionality, the environment in which it is used, comfort, aesthetics, emotional needs, and evaluations by others. Although benefiting from assistive technology (AT), visually impaired people may attract unwanted attention from bystanders when using assistive devices that disclose information about their disability in public areas. Feeling stigmatized or under immense psychological pressure leads to abandonment or refusal to use an assistive device. Coupled with the fact that many assistive devices focus on the production of technology-based tools and lack consideration for the practical needs and problems users encounter in their daily lives, users frequently experience aesthetic and emotional discomfort beyond usability and accessibility [70]. Profita found that many users would hide their devices or divert them to other uses to make them appear less assistive [4]. Alternatively, they may choose to use mainstream technology (e.g., iPhones) rather than highly specialized devices, in order to avoid unwanted attention.

For the community, public concerns about wearable assistive devices for the visually impaired center on personal privacy issues. With the development of computer vision technology, many wearable assistive devices for the visually impaired are fitted with always-on cameras that may trigger negative social comments. Denning et al. report that bystanders do not want to be recorded without their permission or knowledge [75]. Even in extreme cases, negative reactions to the use of wearable computers can lead to censorship or even bans on these devices [76], such as with Google Glass in some public places. Past studies [4,76] showed that bystanders have a more positive view of a device when they are aware of additional information about the device’s use for assistive purposes or the environment in which it is used. Paradoxically, the desire of people who are visually impaired not to disclose information about their disability in public places conflicts with the desire of bystanders to be informed about assistive devices, requiring researchers to think about how to balance the conflicting interests of the two in the design stage. Profita suggested that more generic images or symbols of disability can be used, incorporating information in the terminal that turns features on and off and are disclosed at the user’s discretion, balancing the needs of both parties’ interests in conjunction with societal norms and policies, and including changes in the design or modifications to the software of the assistive tools themselves [4].

In addition, existing research on wearable assistive devices for the visually impaired focuses more on the design of replacements for the functional deficiencies of the users, neglecting the other needs of people who are visually impaired, which go beyond functionality, such as the need for aesthetics and emotions. Although people with disabilities are becoming more prevalent in social and professional life, research regarding fashion item-related motivations and the demands of consumers with disabilities remains limited [77]. Past studies [78,79] found that visually impaired people show high interest in their visual appearance and endeavor to find products that go beyond technology-based usability to suit their preferences. The authors of [70] pointed to the more specific needs of people who are visually impaired, emphasizing that they are very sensitive to the materials and finishes of objects and will assess the aesthetic quality of a product’s appearance and shape through their sense of touch. As a result, the visually impaired may express dissatisfaction with the rough, non-smooth appearance of a device and the lack of straightforward disclosure of disability information. In addition, they would like to be able to assess the level of contamination and manage the maintenance of their wearable items themselves, eliminating the assumption that VIPs neglect personal wear maintenance. Profitaz noted that personalizing the appearance of a device (i.e., bespoke aesthetics) helps to counteract the stigma associated with the use of assistive devices and inspires users to express their individuality by displaying their interests externally [4]. At the same time, understated designs or looks that reflect the mainstream and that are fashionable are perceived as appealing to users.

**Figure 14:**
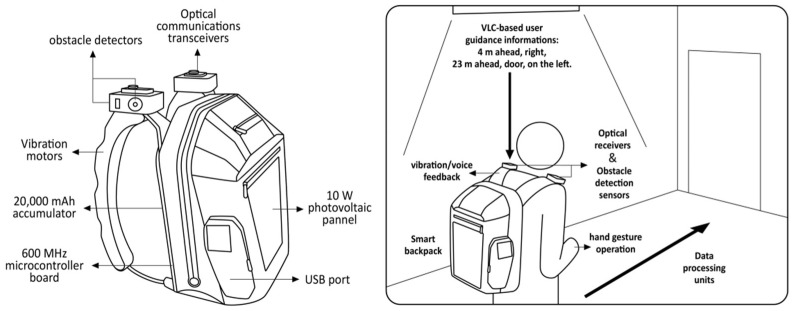
VLC-based assistive system for people who are visually impaired [80].

In recent years, increasing research has focused on the aesthetic and emotional needs of the visually impaired with more consideration given to the user’s experience. The new VLC smart backpack introduced in [80] is a typical example; VLC technology is often applied to accessibility infrastructure to assist people who are visually impaired in indoor navigation, as shown in Figure 14. The prototype was centralized in a backpack, avoiding not only drawing the attention of bystanders but also emphasizing the user’s impairment. The smart backpack was able to convert light from indoor lighting system data into audio or haptic information that can be perceived by the visually impaired. Users request location information for different points of interest through gesture recognition, which, combined with obstacle detection and vibrotactile feedback on the backpack, enhances their mobility and safety in unfamiliar public places. Aziz et al. proposed an affective design model [81] specifically tailored to visually impaired users by combining a triangulation methodology (ITM) and expert assessment to ensure a holistic interactive experience for users who are visually impaired by emphasizing affective interactions, emotions, meaningful delivery, confidence, social contact, cognitive engagement, and curiosity. Kim et al. proposed that VIPs should be designed taking into account social factors, consumers’ emotions, attitudes, and behaviors, considering their impact when making decisions regarding form, materials, details, and other design factors [70]. They used an interdisciplinary study centred on the design process in their research, aiming to integrate different perspectives, foster creativity, and provide holistic solutions while taking wider implications into account, including sustainability, social ethics, and equity. Ortiz-Escobar et al. also support the positive impact of adopting interdisciplinary research, arguing that it reduces the methodological shortcomings observed in today’s literature and helps researchers to develop better designs that meet the social, physiological, cultural, and technological needs of target users [82]. At the same time, they expressed their disapproval of the current approach that seems to be in vogue, arguing that users should be more involved in the design and development process, rather than only in the testing of the final product.

## 4. Discussion

This study presents a bibliometric analysis of research on wearable assistive devices for the visually impaired using CiteSpace. It addresses the following questions: How many studies on wearable assistive devices for the visually impaired were published in WoS over the last decade? Which journals have had the greatest influence? Which authors have been the most active? Is there a core group of authors? Which studies are the most classic in the field? Which research topics and trends have been most prevalent over the last decade? Because of the long cycle of data collection and compilation, as well as the time required for new research to disseminate, bibliometric analysis is able to identify long-term research hotspots and trends, but it is difficult to reflect emerging areas of research and innovation in a timely manner. Therefore, we reviewed the newly published research in the literature based on keywords and clustering results analysed by bibliometrics and found that wearable assistive devices for visually impaired persons based on sensory substitution technology have emerged in recent years with many new innovations and discussions of issues that can be summarised in the following three manners: sensory substitution technology for wearable assistive devices for the visually impaired, the application of smart materials, and research on the issue of the conflicting interests of the individual and society. Some of these issues require interdisciplinary research, and there is a paucity of review articles. Consequently, the second half of this study is centred on these three directions, with the aim of providing inspiration and suggestions for future research.

### 4.1. Summary of the Results of the Bibliometric Analysis

Through a bibliometric analysis, we found that the research on wearable assistive devices for the visually impaired over last decade is characterized as cross-scientific and results in a combination of academia and practice. The growth of research in 2014–2018 was driven by the development of technologies such as intelligent navigation systems and obstacle detection. From 2018–2023, there has been a decline in research enthusiasm, but overall it is stable, possibly due to a gradual saturation of research in this area or a shift in research focus to other directions. Collaborative networks in this research area are fragmented, less central and mostly small-scale, and collaboration among teams is lacking, insufficient. Authoritative collaborators have not yet emerged, and research in this field is still in a discrete state. Keyword co-occurrence and clustering analyses reveal that research in this field tends to develop towards intelligent and wearable technologies.

### 4.2. Optimizing Haptic and Auditory Information Transmission While Multitasking

In recent years, wearable assistive devices for the visually impaired have made significant advances in haptic and auditory feedback technology, but there are still some technical bottlenecks. Tactile feedback technology, especially vibrotactile feedback, can effectively convey navigation information and simple environmental feedback. However, the information capacity of tactile devices is limited [83], especially in complex situations, such as multi-tasking environments, where it is still a challenge to effectively convey information with limited sensory input [84]. Current vibrotactile devices mostly rely on a single feedback mode, which to some extent limits their adaptability to complex environments or tasks. There are also issues with device comfort and learning adaptability, and users need to train for a long time to get used to and effectively understand the feedback content. Electromechanical haptic feedback devices have made progress in reducing cognitive load and improving perception. For example, devices based on pneumatic control or servomotors can use human proprioception to achieve more natural feedback [85], but these devices typically have high power consumption, high noise, and are large and heavy, which limits their widespread use. In addition, the comfort and convenience [86] of the device remains a key design challenge. The application of smart materials, such as shape memory alloys and flexible sensors, has already provided some solutions. By using lighter, more flexible and more efficient materials, future haptic feedback devices could achieve breakthroughs in terms of size, power consumption, and comfort.

The following recommendations are proposed for the above research problems and possible solutions: 1. Increase the capacity of haptic feedback and multimodal integration: Given the limited information capacity of haptic feedback, multimodal fusion of haptic vibration with other forms of feedback should be explored to meet the needs of multitasking in complex environments. Existing research has shown that combining haptic feedback with visual and auditory information can improve the accuracy and efficiency of users’ perception of environmental information [87]. It is recommended that future research considers the synergy between different senses when designing prototypes, reducing sensory conflict in multitasking situations and optimizing the effectiveness of information transfer. 2. Optimize device design to improve comfort and wearability: Use new materials such as flexible materials and shape memory alloys to improve the bulky, noisy and power-hungry problems of existing electromechanical haptic devices, making them lighter and more adaptable. User experience research should also pay more attention to the comfort, convenience and long-term wearability of the device, and help to optimize the design through comprehensive evaluation. 3. Usability research in multitasking scenarios: Currently, most studies only test the effectiveness of haptic or auditory feedback devices in single-tasking scenarios and lack in-depth research on device performance in multi-tasking environments. Future research needs to extend to complex multitasking scenarios and combine psychophysical experiments to determine the optimal combination of different forms of feedback in multitasking environments [88]. In addition, user experience testing should be conducted in real-world environments, including aspects such as cognitive load, attention allocation, ease of use and overall satisfaction, to ensure the effectiveness and usability of devices in multi-tasking scenarios. 4. Investigate AI and adaptive feedback mechanisms: With the development of artificial intelligence technology, the application of AI assistants in wearable assistive devices will become an important way to enhance the intelligence level of devices. Future research should combine AI voice assistants and adaptive feedback mechanisms to enable devices to dynamically adjust their feedback methods [89] based on user behaviour and environmental changes, thereby improving the personalized service [90] level of the device. In addition, customized designs based on user needs and preferences will help improve the learning adaptability of the device and user acceptance.

### 4.3. Advancing the Cross-Disciplinary Application of Smart Materials in Wearable Devices

As the limitations of traditional electronic materials become increasingly apparent, smart materials offer new opportunities for the next generation of wearable assistive devices for the visually impaired, particularly in terms of mechanical flexibility, transparency and integration [91,92], which have great advantages. In particular, smart materials, such as the aforementioned shape memory alloys and electronic skin, have shown great potential in terms of haptic feedback and flexible sensors, improving wearer comfort and device adaptability. However, the practical application of smart materials still faces several technical challenges. Firstly, the interaction between the material and the skin [93] (e.g., sweat, oil) can affect the long-term stability and comfort [94] of the device. In addition, although electrostatic actuators and conductive textiles are excellent in terms of comfort and flexibility, their response speed and accuracy [65] still need to be improved. In terms of sustainability, although environmentally friendly polymer materials have been investigated, their cost and reliability in mass production and practical applications [95] need to be further verified.

The following research recommendations are therefore proposed 1. Multi-functional integration and material optimization: Future research should focus on how to optimize material properties, such as improving conductivity, flexibility and durability, and combine multifunctional integration to meet the needs of lightweight and high performance devices. In particular, for haptic feedback devices, how to balance comfort and high precision feedback remains an urgent problem to be solved. 2. Interdisciplinary cooperation and innovative design: Given the complexity of smart materials, it is recommended to promote interdisciplinary collaboration in fields such as materials science, mechanical engineering, electronics and biomedicine to accelerate the application and iteration of new materials in wearable visual aids. 3. User experience and sustainability assessment: The development of smart materials should consider not only the functionality of the materials, but also the long-term user experience of the device, such as durability, comfort and adaptability to the skin. In addition, the production process and cost of sustainable materials should be explored to reduce the environmental footprint of the device and ensure its affordability and feasibility.

### 4.4. Balancing the Interests of the Individual and Society

In response to public concerns about privacy and security with wearable assistive device cameras, this study suggests that devices consider using algorithms that can blur faces or encrypt data. Processing the data locally instead of sending it to the cloud allows the user to control whether facial recognition functions are turned on or off to balance the need of individuals to benefit from assistive devices with the need to protect the public’s privacy. Consideration also needs to be given to curbing the illegal collection of others’ private information through social norms and policies, and greater social awareness of new technologies is needed to mitigate negative public perceptions due to lack of understanding.

For people who are visually impaired that do not wish to disclose information about their disability, which conflicts with the desire of bystanders to be informed about assistive devices, this study suggests that disability information can be conveyed using more generic or symbolic graphics or text, with disclosure left to the user’s discretion or displayed only to a specific group of people (e.g., service personnel at a public facility) to avoid assumptions about the disability. Researchers can also consider making the appearance of the device as discreet as possible to avoid this problem, if it is not actually designed function to invade the privacy of others with its assistive features.

Wearable assistive devices for the visually impaired should fully consider their characteristics and needs and, on the basis of usability and safety, they should be as tactile as possible, with aesthetic qualities appreciated by its users. In addition, thanks to the development of smart materials, wearable assistive devices for the visually impaired can be designed in the form of lightweight, body-fitting clothing and accessories. As a result, wearable assistive devices for the visually impaired may have decorative and fashionable attributes that go beyond assistive functions. In the future, researchers may need to consider wearable assistive devices as part of a person’s attire, taking into account their fashion and aesthetic preferences, level of independent daily management and maintenance, customization, and personalized expression. Physical and psychological differences between people who are visually impaired and those who are not can lead to significant differences in their aesthetic and emotional needs, even though, among people with visual impairment, differences in needs also exist due to differences in gender, age, and cultural backgrounds. As there is limited research on the motivations and needs of visually impaired people for aesthetic fashion consumption, this study encourages researchers to consider supplementing the research in this area.

The adoption of wearable assistive devices is influenced by complex factors, including sociocultural considerations (e.g., stigma), personal values, and motivations associated with AT usage [4]. We found that the acceptance of new technologies or devices by people who are visually impaired may change over time. For example, before Google Glass was popular, the visually impaired preferred to use traditional devices, such as guide canes, to avoid attracting too much attention in public places. However, with the popularity of Google Glass, an increasing number of people are willing to use smart glasses, praising their performance and sophisticated and lightweight appearance, as if they were wearing a popular piece of technology rather than an assistive device for a disability. We also found that many studies only invite people with visual impairments to participate in testing the usability of the final product, and very few invite them to participate in the design and development processes. This situation may easily lead to the final product not meeting the real everyday needs of the visually impaired or being discarded because of social acceptance issues. This study suggests that it is necessary for researchers to consider the impact of current sociocultural factors or those predicted over the next few years; to invite people with visual impairments or researchers from other fields to participate in the design and development processes; and to increase testing of their social acceptance to avoid the design being affected by the limitations of single-domain knowledge and perceptions.

### 4.5. Two Future Developments

Summarizing the research and discussion of the three directions above, we believe that. in the future, the design of wearable assistive devices for the visually impaired based on sensory substitution technology will move towards a comprehensive solution that is lightweight, energy efficient, highly integrated, highly intelligent, takes full account of the aesthetic and emotional needs of the user, and is easy to maintain and manage. We believe that there are two directions with great potential that researchers should further explore and expand. The first is the low-cost pursuit of efficiency and independence, and the second is the high-cost pursuit of high-quality services and in-depth coupling with the construction of barrier-free infrastructure. These two directions are proposed in consideration of the uneven development among different regions and populations in terms of economy, technology, and accessibility infrastructure construction.

The low-cost, efficiency-seeking, and stand-alone direction considers the simplification and diffusion of wearable assistive devices for the visually impaired so that more people can afford to use these devices to meet their basic daily living needs. It requires simpler designs, more economical materials, and the optimization of existing technologies to reduce production and usage costs. Specifically, a simple assistive device based on a user’s existing smartphone can be developed, connecting via Bluetooth and other wireless technologies, using the camera, sensors, and computing power of the mobile phone or combined with a cloud processor to process the collected environmental data, and guiding the user via haptic feedback or providing voice prompts through a headset. Assistive devices can be integrated into belt pouches, waistcoats, and jackets. Not only is it easy to place and operate a smartphone, but the user can effectively perceive the haptic feedback in comfort. This allows for an unobtrusive, everyday appearance that does not draw too much attention from onlookers. Haptic feedback devices can consider using vibrating actuators combined with low-cost new material components (e.g., incorporating SMA wires into haptic actuators) to provide richer haptic feedback information. They are inexpensive, with easy to sense feedback, efficient with low power consumption, and easily integrated into textiles.

The pursuit of high-quality services at high cost and deeply integrated with accessibility infrastructure suggests the use of existing mature devices and platforms. High-performance wearable assistive devices for the visually impaired will be integrated in depth with smart cities and accessibility infrastructure to provide high-precision navigation and environmental sensing capabilities. Meanwhile, seamless integration with surrounding smart infrastructure (bus stops, traffic lights, indoor lighting systems, indoor navigation systems, etc.) can be achieved to improve the mobility and safety of visually impaired people. Google Glass is an ideal device with highly integrated functions, an elegant and lightweight appearance, and high social acceptance. Its powerful sensors, computing power, and various applications not only help people who are visually impaired to complete basic tasks, such as navigation and obstacle detection, but feature AI-based voice assistants combined with a variety of wearable haptic feedback devices to achieve real-time access to the Internet for shopping, learning, entertainment, and other more diverse needs. As this direction pursues high-quality services, wearable haptic feedback devices can apply more intelligent materials and wearable soft technologies. In the achievement of high functional efficiency, the “fashion” aspect and the maintenance and management needs of the wearable device should be further considered to satisfy the higher aesthetic and emotional needs of the visually impaired.

## 5. Conclusions

Wearable assistive devices for the visually impaired are an important aspect of assistive technology and robotics research. This study is a bibliometric-based narrative review of the research on wearable assistive devices for the visually impaired over the past decade, and it provides researchers with insights into the current status of research and the trends and hotspots in this field. Wearable assistive devices based on sensory substitution technology are a prominent direction of research among the hotspots in this field, giving rise to numerous innovative interdisciplinary designs and studies. It was found that advances in sensory substitution technologies have provided more innovative feedback methods and feedback interfaces for wearable assistive devices for the visually impaired, offering promising solutions for achieving accessibility and independence in the daily lives of the visually impaired. The application of smart materials in this field has been particularly effective, helping to overcome the limitations of traditional electronic materials and enabling these wearable assistive devices to be thin, lightweight, low power, and comfortable to wear, fitting the body’s curves. However, acceptance by individuals and society is becoming an important issue for the adoption of wearable assistive devices for the visually impaired, and balancing the interests of both parties is key to solving this challenge. In turn, the following recommendations for the design of wearable assistive devices are proposed: (1) optimize the transmission of haptic and auditory information while multitasking; (2) advance research on smart materials and foster cross-disciplinary collaboration among experts; and (3) balance the interests of individuals and society. In addition, the following two possible directions for future development are proposed: low-cost pursuit of efficiency and independence, and high-cost pursuit of high-quality services that are deeply integrated with accessibility infrastructure.

## Figures and Tables

**Table 1:** Top 10 co-citation journals of the studied publications from 2014 to 2024.

Rank	Number of Articles Published	Centrality	Year	Journal
1	205	0.03	2014	Lecture Notes in Computer Science
2	168	0.07	2014	Sensors
3	127	0.05	2014	IEEE Conference on Computer Vision and Pattern Recognition Proceedings
4	85	0.11	2014	IEEE Transactions on Systems, Man, and Cybernetics: Systems
5	76	0.06	2014	IEEE International Conference on Robotics and Automation
6	75	0.02	2014	IEEE Transactions on Pattern Analysis and Machine Intelligence
7	74	0.02	2019	IEEE Access
8	66	0.04	2015	IEEE Transactions on Neural Systems and Rehabilitation Engineering
9	64	0.06	2014	Journal of Visual Impairment and Blindness
10	63	0.05	2015	Public Library of Science ONE

**Table 2:** Top 10 authors of the studied publications from 2014 to 2024.

Rank	Number of Articles Published	Author
1	20	Kaiwei Wang
2	18	Kailun Yang
3	14	Ruiqi Cheng
4	10	Jinqiang Bai
5	8	Weijian Hu
6	5	Ningbo Long
7	5	Leah Findlater
8	5	Liang-Bi Chen
9	5	Wan-Jung Chang
10	4	Lee Stearns

**Table 3:** Top 10 co-cited references of the studied publications from 2014 to 2024.

Rank	Freq	Centrality	Year	Title
1	35	0.02	2017	*Magnitude, Temporal Trends, and Projections of the Global Prevalence of Blindness and Distance and Near Vision Impairment: A Systematic Review and Meta-Analysis*
2	33	0.16	2016	*Navigation Assistance for the Visually Impaired Using RGB-D Sensor With Range Expansion*
3	28	0.05	2017	*Sensor-Based Assistive Devices for Visually Impaired People: Current Status, Challenges, and Future Directions*
4	24	0.06	2017	*Enabling Independent Navigation for Visually Impaired People Through a Wearable Vision-Based Feedback System*
5	20	0.11	2017	*Smart Guiding Glasses for Visually Impaired People in Indoor Environments*
6	19	0.08	2018	*Safe Local Navigation for Visually Impaired Users With a Time-of-Flight and Haptic Feedback Device*
7	18	0.01	2018	*Unifying Terrain Awareness for the Visually Impaired Through Real-Time Semantic Segmentation*
8	18	0.09	2016	*Expanding the Detection of Traversable Area With RealSense for the Visually Impaired*
9	17	0.09	2021	*World Health Organization, 2021, Blindness and Vision Impairment*
10	16	0.04	2019	*Vision-Based Mobile Indoor Assistive Navigation Aid for Blind People*

**Table 4:** Top 15 keywords by frequency for the publications studied from 2014 to 2024.

Rank	Count	Centrality	Year	Keywords
1	151	0.3	2014	visually impaired
2	73	0.21	2014	computer science
3	70	0.39	2014	assistive technology
4	48	0.21	2014	instruments and instrumentation
5	31	0.1	2015	computer vision
6	28	0.06	2015	wearable device
7	26	0.08	2014	obstacle detection
8	22	0.11	2015	deep learning
9	21	0.09	2014	object recognition
10	18	0.08	2014	obstacle avoidance
11	17	0.14	2018	materials science
12	17	0.09	2016	ultrasonic sensor
13	15	0.09	2016	assistive device
14	15	0.02	2014	object detection
15	13	0.09	2016	wearable system

**Table 5:** Top 15 keywords by centrality for the studied publications from 2014 to 2024.

Rank	Centrality	Count	Year	Keywords
1	0.39	70	2014	assistive technology
2	0.3	151	2014	visually impaired
3	0.21	73	2014	computer science
4	0.21	48	2014	instruments and instrumentation
5	0.14	17	2018	materials science
6	0.11	22	2015	deep learning
7	0.11	7	2015	low vision
8	0.1	31	2015	computer vision
9	0.09	21	2014	object recognition
10	0.09	17	2016	ultrasonic sensor
11	0.09	15	2016	assistive device
12	0.09	13	2016	wearable system
13	0.08	26	2014	obstacle detection
14	0.08	18	2014	obstacle avoidance
15	0.08	12	2015	wearable technology

**Table 6:** Classification of keyword clusters for the studied publications from 2014 to 2024.

Cluster ID	Size	Silhouette	Year	Top Terms (LSI)
0	46	0.796	2018	computer science; object recognition; assistive technology; real-time systems; impaired people/smart glasses; obstacle avoidance; assistive devices; collision avoidance; legged locomotion
1	31	0.946	2017	assistive technology; computer science; wearable computers; navigation device; wearable system/electronic travel aids; computer vision; visual impairment; patent analysis; vision impairment
2	25	0.892	2018	wearable technology; sport assistance; augmented reality; impaired people; navigation aids/symptom monitoring; medical informatics; cross-section interview; health data; mhealth technology
3	23	0.921	2017	assistive technology; computer science; wearable device; obstacle detection; electronic travel aids/visual impairment; information technology; daily living; visual odometry; stair detection
4	22	0.903	2017	object detection; haar transformation; positional analysis; cmos camera; object recognition/visual impairment; convolutional neural; wearable assistive system; ground segmentation; three-dimensional displays
5	20	0.87	2017	obstacle detection; wearable device; assistive device; Bluetooth module; navigation aid/computer science; mobility aid; blind assistive; feature fusion; multilayer gru
6	20	0.79	2018	ultrasonic sensor; audio feedback; visual impairment; object recognition; smart system/object recognition; raspberry pi; computer science; blind people; navigation device
7	19	0.919	2018	wearable system; computer vision; navigational aid; indoor navigation; radar detection/handicapped aids; radar detection; signal processing algorithm; millimeter wave radar; frequency-modulated continuous wave radar system
8	17	0.776	2018	human-computer interaction; computer science; wearable computing; augmented vision; sensory augmentation/visual impairments; virtual reality; auditory feedback; assistive wearable devices; collaborative design
9	17	0.851	2022	materials science; other topics; braille typing system; current signal; blindness assistance/fall detection; indoor navigation monitoring; anti-collision system; blind people support; non-contact triboelectric sensor
10	13	0.925	2019	machine learning; wireless sensor networks; heuristic algorithms; sensing systems; assistive technology/positioning systems; visual impairment; user acceptance; vehicles opportunities; cognitive map
11	13	0.807	2019	deep learning; computer science; object detection; mobile edge; wireless communication/assistive technology; crosswalk detection; pedestrian detection; crossing light detection; dead reckoning
12	11	0.893	2023	deep learning; industrial robots; impedance measurement; surface impedance; terrain classification/legged locomotion; current measurement; humanoid robots; mono-filament issues; deep learning
13	5	1	2022	bio-inspired navigation; visual localization; navigation assistive; devices; artificial place cells; artificial grid cells; artificial head; direction cells
14	4	1	2023	viola jones algorithm; haar cascades; opencv; py-yolov5 algorithm; onnx; model; deep neural networks; eigen faces; principal component; analysis; azure maps; octa-polar segmentation; dimensional ratio; similarity; py-tesseract; paddle ocr
15	3	1	2017	wvns; a combinatorial planner; aiming-tracking mechanism; autoregressive; model; dynamic weighted a*

## Data Availability

Not applicable.
